# Current treatment status of IgA nephropathy in Japan: a questionnaire survey

**DOI:** 10.1007/s10157-023-02396-0

**Published:** 2023-08-30

**Authors:** K. Matsuzaki, H. Suzuki, M. Kikuchi, K. Koike, H. Komatsu, K. Takahashi, I. Narita, H. Okada, Committee of Clinical Practical Guideline for IgA Nephropathy 2020

**Affiliations:** 1 Department of Public Health, Kitasato University School of Medicine, Kanagawa, Japan; 2 Department of Nephrology, Juntendo University Urayasu Hospital, Chiba, Japan; 3 Department of Nephrology, Faculty of Medicine, University of Miyazaki, Miyazaki, Japan; 4 Division of Nephrology and Hypertension, Department of Internal Medicine, The Jikei University School of Medicine, Tokyo, Japan; 5 Center for Medical Education and Career Development, Faculty of Medicine, University of Miyazaki, Miyazaki, Japan; 6 Department of Biomedical Molecular Sciences, Fujita Health University School of Medicine, Aichi, Japan; 7 Division of Clinical Nephrology and Rheumatology, Niigata University Graduate School of Medical and Dental Sciences, Niigata, Japan; 8 Department of Nephrology, Saitama Medical University, Saitama, Japan

**Keywords:** IgA nephropathy, Clinical practice guideline, Questionnaire survey, Practice pattern

## Abstract

**Background:**

In 2020, the Committee of Clinical Practical Guideline for IgA Nephropathy (IgAN) revised the clinical practice guidelines. Herein, we conducted a questionnaire survey to assess the potential discrepancies between clinical practice guidelines and real-world practice in Japan.

**Methods:**

A web-based survey of members of the Japanese Society of Nephrology was conducted between November 15 and December 28, 2021.

**Results:**

A total of 217 members (internal physicians: 203, pediatricians: 14) responded to the questionnaire. Of these respondents, 94.0% answered that the clinical practice guidelines were referred to “always” or “often.” Approximately 66.4% respondents answered that histological grade (H-Grade) derived from the “Clinical Guidelines for IgA nephropathy in Japan, 3rd version” and the “Oxford classification” were used for pathological classification. Moreover, 73.7% respondents answered that the risk grade (R-grade) derived from the “Clinical Guidelines for IgA nephropathy in Japan, 3rd version” was referred to for risk stratification. The prescription rate of renin–angiotensin system blockers increased based on urinary protein levels (> 1.0 g/day: 88.6%, 0.5–1.0 g/day: 71.0%, < 0.5 g/day: 25.0%). Similarly, the prescription rate of corticosteroids increased according to proteinuria levels (> 1.0 g/day: 77.8%, 0.5–1.0 g/day: 52.8%, < 0.5 g/day: 11.9%). The respondents emphasized on hematuria when using corticosteroids. In cases of hematuria, the indication rate for corticosteroids was higher than in those without hematuria, even if the urinary protein level was 1 g/gCr or less. Few severe infectious diseases or serious deterioration in glycemic control were reported during corticosteroid use.

**Conclusion:**

Our questionnaire survey revealed real-world aspects of IgAN treatment in Japan.

**Supplementary Information:**

The online version contains supplementary material available at 10.1007/s10157-023-02396-0.

## Introduction

Immunoglobulin A nephropathy (IgAN) is the most common type of chronic glomerulonephritis in Japan. It is a refractory disease, which leads to end-stage renal failure in 30–40% of patients after approximately 20 years [1]. Although various studies have been conducted to date, the use of standard clinical questions in daily clinical practice has not been established because IgAN follows a chronic course with various clinical features. Therefore, accurate medical care for IgAN has not been established.

In 2020, the Research for Intractable Renal Diseases of the Ministry of Health, Labour and Welfare of Japan established the Committee of Clinical Practical Guideline for IgA Nephropathy and published the guideline on its website [2]. Subsequently, a digest version of the guideline was published in 2021 [3]. This guideline explains several treatment options for IgAN and recommends renin–angiotensin system (RAS) blockers and corticosteroids as grade IB (strong recommendation, moderate-quality evidence) based on the results of a systematic review.

Herein, we conducted a questionnaire survey that aimed to assess the extent to which the guidelines were followed in Japan and to reveal several aspects of IgAN treatment.

## Methods

In this survey, a web-based questionnaire was distributed to members of the Japanese Society of Nephrology via subscribed mailing list between November 15 and December 28, 2021. It consisted of questions divided into five sections including: (i) baseline characteristics of the responders (Q1–Q7); (ii) “Evidence-Based Clinical Practice Guideline for IgA Nephropathy 2020 [2]” and “Clinical Guidelines for IgA nephropathy in Japan, 3rd version [4]” (Q8–Q10); (iii) selection of the treatment options in detail (Q11–Q21); (iv) practice pattern of RAS blockers and corticosteroids (Q22–Q27); and (v) factors to determine treatment options (Q28–Q31). These questions were deliberated by the specialists of the committee of the guideline for IgA nephropathy though the peer-review from committee members of other disease guideline. Table 1 presents the questionnaire details. If the respondents were treating patients who were newly diagnosed with IgAN, they answered questions after Q11.

**Table 1:** Details of the questionnaire

	Questions	Answers
Q1	What kind of institute are you affiliated with?	1. University hospital 2. Teaching hospital certificated by Japan Society of Nephrology 3. General hospital 4. Clinic
Q2	Which prefecture is your institution located in?	Respondents selected from a pull-down list consisting of 47 prefectures
Q3	What kind of patients do you mainly treat?	1. Adult patients 2. Child patients
Q4	How many years of experience as a doctor do you have? (including internship)	1. < 5 2. 5– < 10 3. 10– < 20 4. 20≤
Q5	How many beds in your institution where you mainly practice?	1. < 200 2. 200–500 3. ≥ 500
Q6	Approximately how many renal biopsies does your hospital performed in a year at your institution?	1. < 20 2. 20–50 3. ≥ 50
Q7	Approximately how many patients with IgA nephropathy does your hospital diagnosed in a year at your institution?	1. < 10 2. 10–30 3. ≥ 31
Q8	Do you refer to the “evidence-based clinical practice guideline for IgA nephropathy 2020” when treating the patients with IgA nephropathy?	1. Always 2. Often 3. Seldom 4. Never
Q9	Which classification do you use for pathological diagnosis of IgA nephropathy?	1. Histological grade based on the "Clinical Guidelines for IgA nephropathy in Japan, 3rd version” 2. Oxford classification 3. Both 1. and 2., 4. Others
Q10	Which do you refer to for prognosis of patients with IgA nephropathy, the risk classification (RG) based on the “Clinical guidelines for IgA nephropathy, 3rd version” or the International risk-prediction tool (JAMA Intern Med. 2019)?	1. Risk grade based on the "Clinical Guidelines for IgA nephropathy in Japan, 3rd version” 2. International risk-prediction tool 3. Both 1. and 2., 4. Neither 1. and 2
Q11	What percentage of newly diagnosed patients with IgA nephropathy undergo tonsillectomy and steroid pulse therapy?	1. 0–30%, 2. 31–70%, 3. ≥ 71%
Q12	Which situations would you consider the indication for tonsillectomy? (Multiple answers are acceptable)	1. Highly disease activity that require immunosuppressive therapy 2. History of habitual tonsillitis 3. Episodes of gross hematuria after tonsillitis 4. Patient's Wishes 5. Otolaryngology department does not perform the tonsillectomy for patient with IgA nephropathy, thus the tonsillectomy is not usually performed
Q13	If the patients shows the hematuria, what amounts of urinary protein would you indicate that the administration the corticosteroids?	1. ≥ 1 g/gCr (or g/day) 2. 0.5–< 1 g/gCr (or g/day) 3. ≤ 0.5 g/gCr (or g/day)
Q14	If the patients does not show the hematuria, what amounts of urinary protein would you indicate that the administration the corticosteroids?	1. ≥ 1 g/gCr (or g/day) 2. 0.5–< 1 g/gCr (or g/day) 3. ≤ 0.5 g/gCr (or g/day)
Q15	Which drug is used to prevent side effects when corticosteroid therapy for IgA nephropathy is initiated? (Multiple answers are acceptable)	1. Sulfamethoxazole-trimethoprim combination 2. Medications for osteoporosis 3. Proton pump inhibitor (PPI) 4. H2 receptor antagonist (H2 blocker) 5. Antifungal agent 6. Antituberculous drug 7. None of the listed are used
Q16	What is the rate of occurrence of abnormal glycemic control requiring treatment during corticosteroid therapy for IgA nephropathy?	1. ≤ 10% 2. 10–30% 3. 30–50% 4. ≥ 50%
Q17	What is the frequency of obvious infections that requiring antibiotic therapy during corticosteroid therapy for IgA nephropathy?	1. ≤ 10% 2. 10–30% 3. 30–50% 4. ≥ 50%
Q18	How many patients do you use immunosuppressive drugs excluding corticosteroids in a year?	1. None 2. 1–< 5patients 3. 5–< 10 patients 4. ≥ 10 patients
Q19	If you use the immunosuppressive agents excluding corticosteroids, which drug is the first-line agent?	1. Mizoribine 2. Cyclophosphamide 3. azathioprine 4. Cyclosporine 5. Mycophenolate mofetil
Q20	What is the rate of using the antiplatelet agents (e.g., dipyridamole, zirazep hydrochloride) for IgA nephropathy?	1. Seldom 2. Less than 1/3 3. Almost 1/3–2/3 4. 2/3 or more
Q21	What is the rate of using the n-3 fatty acid for IgA nephropathy?	1. Seldom 2. Less than 1/3 3. Almost 1/3–2/3 4. 2/3 or more
Q22	In case of the UPCR > 1 g at the diagnose of IgA nephropathy, what is the prescription rate of RAS blocker?	1. Seldom 2. Less than 1/3 3. Almost 1/3–2/3 4. 2/3 or more
Q23	In case of the UPCR 0.5-1 g at the diagnose of IgA nephropathy, what is the prescription rate of RAS blocker?	1. Seldom 2. Less than 1/3 3. Almost 1/3–2/3 4. 2/3 or more
Q24	In case of the UPCR < 0.5 g at the diagnose of IgA nephropathy, what is the prescription rate of RAS blocker?	1. Seldom 2. Less than 1/3 3. Almost 1/3–2/3 4. 2/3 or more
Q25	In case of the UPCR > 1 g at the diagnose of IgA nephropathy, what is the prescription rate of corticosteroids?	1. Seldom 2. Less than 1/3 3. Almost 1/3–2/3 4. 2/3 or more
Q26	In case of the UPCR 0.5–1 g at the diagnose of IgA nephropathy, what is the prescription rate of corticosteroids?	1. Seldom 2. Less than 1/3 3. Almost 1/3–2/3 4. 2/3 or more
Q27	In case of the UPCR < 0.5 g at the diagnose of IgA nephropathy, what is the prescription rate of corticosteroids?	1. Seldom 2. Less than 1/3 3. Almost 1/3–2/3 4. 2/3 or more
Q28	When you prescribe RAS blocker for patients with IgA nephropathy, how important do you consider following factors (a. eGFR, b. urinary protein, c. histological findings, d. degree of the hematuria, e. blood pressure, f. age)?	(For items a through f) 1. Very important, 2. Some importance, 3. Neither important nor unimportant, 4. Not very important, 5. Unimportant
Q29	When you prescribe corticosteroids for patients with IgA nephropathy, how important do you consider following factors (a. eGFR, b. urinary protein, c. histological findings, d. chronic lesion in the pathological findings, e. degree of the hematuria, f. blood pressure, g. age)?	(For items a through g) 1. Very important, 2. Some importance, 3. Neither important nor unimportant, 4. Not very important, 5. Unimportant
Q30	When you perform tonsillectomy and steroid pulse therapy for patients with IgA nephropathy, how important do you consider following factors (a. eGFR, b. urinary protein, c. histological findings, d. degree of the hematuria, e. blood pressure, f. age)?	(For items a through f) 1. Very important, 2. Some importance, 3. Neither important nor unimportant, 4. Not very important, 5. Unimportant
Q31	When determining the remission of IgA nephropathy, do you use the “remission criteria for IgA nephropathy”?	1. Yes 2. No

## Results

A total of 217 members (203 internal physicians and 14 pediatricians) responded to the questionnaire survey.

### Adherence to clinical guidelines

**Fig. 1:**
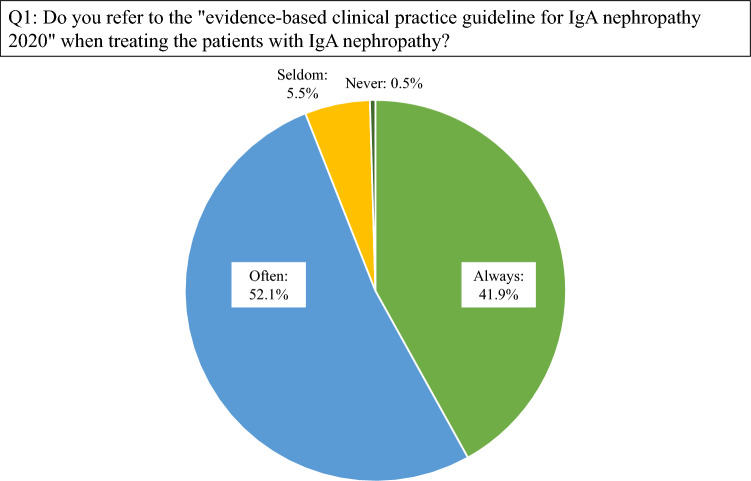
Practical use of the “evidence-based clinical practice guideline for IgA nephropathy 2020.” Of the respondents, 94.0% have answered that this guideline is referred to “always” or “often”

204 respondents (94.0%) answered that “evidence-based clinical practice guideline for IgA nephropathy 2020” was referred to “always” or “often” (Fig. 1). Regarding the pathological classifications, 144 respondents (66.4%) answered that both the H-grade derived from “Clinical Guidelines for IgA nephropathy in Japan, 3rd version [4]” and “Oxford classification [5]” were used. Additionally, 60 respondents (27.6%) answered that only the H-grade derived from the “Clinical Guidelines for IgA nephropathy in Japan, 3rd version” was used. Regarding risk stratification, 160 respondents (73.7%) referred to only the R-grade derived from the “Clinical Guidelines for IgA nephropathy in Japan, 3rd version.” Furthermore, 43 respondents (19.8%) referred to both the “Clinical Guidelines for IgA nephropathy in Japan, 3rd version” and “International prediction tool [6].” To determine the remission of IgAN, 140 respondents (79.5%) used the remission criteria proposed by Suzuki et al. [7].

### RAS blocker

**Fig. 2:**
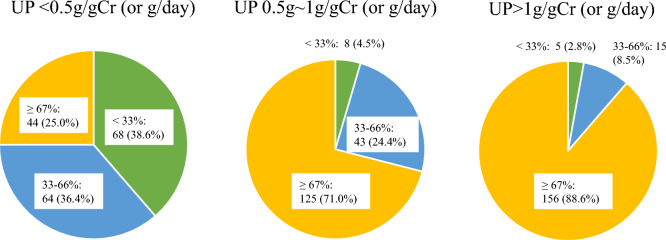
Prescription rate of RAS blockers according to the amount of proteinuria. The prescription rate of RAS blockers increases with elevated proteinuria. Only 44 respondents (25.0%) prescribe RAS blockers for 67% or more in cases of proteinuria < 0.5 g/day

The prescription rate of RAS blocker according to urinary protein levels is shown in Fig. 2. One hundred fifty-six respondents (88.6%) prescribed RAS blocker for 67% or more cases of urinary protein level > 1 g/day, 125 respondents (71.0%) prescribed RAS blocker for 67% or more cases of urinary protein levels of 0.5–1.0 g/day. Conversely, only 44 respondents (25.0%) prescribed RAS blocker for 67% or more cases of urinary protein level < 0.5 g/day. Regarding prescription of RAS blocker, 148 respondents (84.1%) answered “more important” with eGFR, 124 respondents (70.5%) with histological findings, 169 respondents (96.0%) with blood pressure, and 127 respondents (72.2%) with age (Supplementary Fig. 1).

### Corticosteroids

**Fig. 3:**
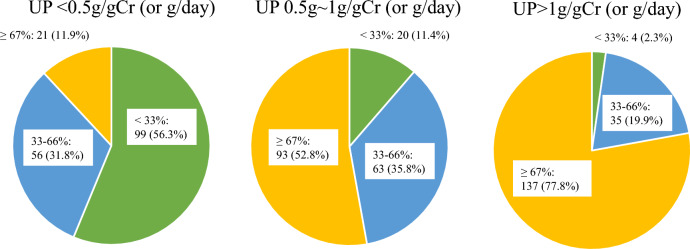
Prescription rate of corticosteroids based on the amount of proteinuria. The prescription rate of corticosteroids well associated with the amount of proteinuria

The prescription rate of corticosteroids according to urinary protein levels is shown in Fig. 3. One hundred thirty-seven respondents (77.8%) prescribed corticosteroids for 67% or more cases of urinary protein level > 1 g/day, 93 respondents (52.8%) prescribed corticosteroids for 67% or more cases of urinary protein level of 0.5–1.0 g/day. Conversely, only 21 respondents (11.9%) prescribed corticosteroids for 67% or more cases of urinary protein level < 0.5 g/day. Regarding prescription of corticosteroids, 153 respondents (86.9%) answered “more important” with eGFR, 175 respondents (99.4%) with histological findings, 127 respondents (72.2%) with degree of hematuria, and 149 respondents (84.7%) with age (Supplementary Fig. 2). The frequency of concomitant drug use with corticosteroids is shown in Table 2. The common concomitant drugs at the start of corticosteroid use were anti-osteoporosis drugs (154 respondents, 87.5%), proton pump inhibitors (146 respondents, 83.0%), and sulfamethoxazole/trimethoprim (127 respondents, 72.2%). Table 3 shows the proportion of patients with impaired glucose tolerance and obvious infections due to corticosteroid therapy. One hundred fifty-one respondents (85.8%) answered that impaired glucose tolerance requiring treatment after corticosteroid administration occurred in less than 30% of the cases. Similarly, 166 respondents (94.3%) answered that infectious diseases requiring antimicrobial treatment occurred in less than 10% of the cases.

**Table 2:** Frequency of the concomitant drugs during treatment with corticosteroids

*n* = 176	
Medications for osteoporosis	154 (87.5%)
Proton pump inhibitor (PPI)	146 (83.0%)
H2 receptor antagonist (H2 blocker)	23 (13.1%)
Sulfamethoxazole-trimethoprim combination	127 (72.2%)
Antifungal agent	8 (4.5%)
Antituberculous drug	5 (2.8%)
None of the listed are used	6 (3.4%)

**Table 3:** Proportions of the serious deterioration of glycemic control and severe infectious disease requiring antibiotics

	(*n* = 176)
Seriously deterioration of glycemic control	
≤ 10%	74 (42.0%)
10–30%	77 (43.8%)
30–50%	24 (13.6%)
≥ 50%	1 (0.6%)
Severe infectious disease requiring antibiotics	
≤ 10%	166 (94.3%)
10–30%	10 (5.7%)
30–50%	0 (0%)
≥ 50%	0 (0%)

### Tonsillectomy and steroid pulse therapy

**Fig. 4:**
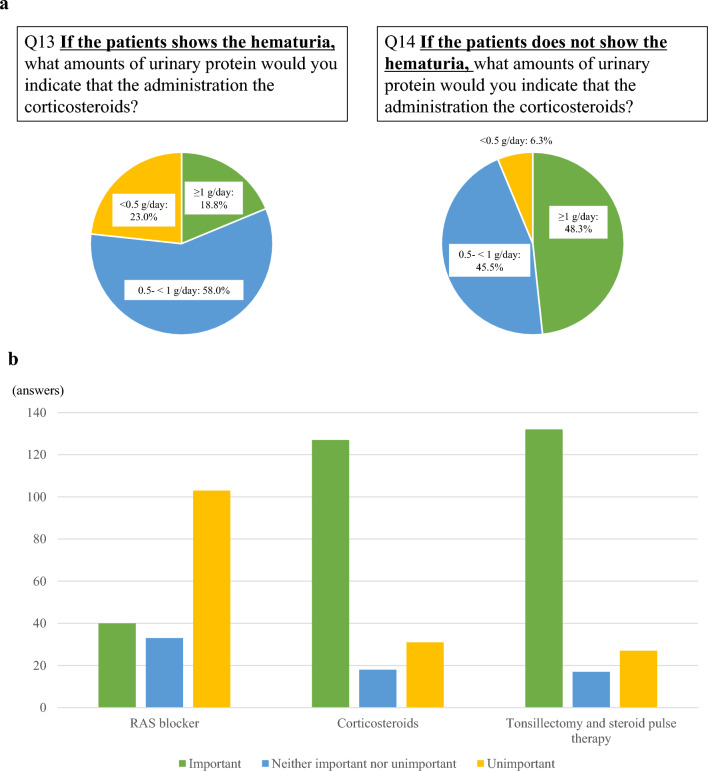
**a** Differences in the indication for corticosteroids based on presence or absence of hematuria. There was a trend that presence of the hematuria caused active use of corticosteroids even in case of lower amount of proteinuria. **b** Importance of hematuria in each treatment option. Japanese physicians emphasize hematuria as an important factor to determine the indication of corticosteroids and TSP

In patients with newly diagnosed IgAN, 76 respondents (35.8%) reported performing tonsillectomy with steroid pulse (TSP) therapy in 71% or more cases. The common indications for tonsillectomy were disease activity (147 respondents, 83.5%), history of habitual tonsillitis (136 respondents, 77.3%), and gross hematuria after upper respiratory tract infection (127 respondents, 72.2%). The amount of proteinuria considered when administering corticosteroids varied according to the presence or absence of hematuria (Fig. 4a). Furthermore, Japanese physicians emphasize hematuria as an important factor to determine the indication of corticosteroids and TSP (Fig. 4b). Regarding TSP therapy, 149 respondents (84.7%) answered “very important” with eGFR, 163 respondents (92.6%) with histological findings, 132 respondents (75.0%) with degree of hematuria, and 154 respondents (87.5%) with age (Supplementary Fig. 3).

### Other drugs including immunosuppressants

One hundred fifty-three respondents (86.9%) reported the use of immunosuppressive drugs other than corticosteroids in fewer than five cases per year. Of these 153 respondents, 113 (64.2%) reported only one case every few years. Sixty-nine respondents (39.2%) prescribed antiplatelet agents in less than 33% of cases, whereas 47 (26.7%) respondents did not prescribe them. Ninety-nine respondents (50.6%) prescribed n-3 fatty acids in less than 33% of cases, whereas 67 respondents (38.1%) did not prescribe them.

## Discussion

This study revealed three notable findings. First, the Japanese nephrologists adhered to the guideline recommendations. Second, corticosteroids were relatively safe in Japan because the Japanese nephrologists carefully checked for side effects and used appropriate dosage. Third, the Japanese nephrologists recognized hematuria as a pivotal factor to determine the indication of corticosteroids and TSP.

Pathological examination is essential for establishing a definitive diagnosis of IgAN. Currently, there are two major pathological classifications: the H-grade according to the “Clinical guidelines for IgA nephropathy in Japan, 3rd version [4],” and the “Oxford classification [5].” The H-grade allows for comprehensive assessment of histological severity owing to the use of a lumped system, and the Oxford classification allows for detailed histological findings owing to the use of a split system. Our study demonstrated that 66.4% of the respondents referred to both the H-grade and the Oxford classification. This result was similar to that of a questionnaire survey conducted among councilors of the Japanese Society of Nephrology in 2018 [2]. However, regarding risk stratification, 73.7% of the respondents referred to only the R-grade derived from the “Clinical Guidelines for IgA nephropathy in Japan, 3rd version.” The R-grade determines the risk of end-stage kidney disease (ESKD) by combining the H-grade and clinical severity; consequently, it is easy to use in daily clinical practice. These results suggest that Japanese nephrologists should emphasize the pathological findings and attempt to establish the prognosis of IgAN.

According to the guidelines, RAS blocker are recommended as grade IB (strong recommendation, moderate-quality evidence) based on a systematic review. Several clinical trials on RAS blocker for IgA nephropathy suggested the efficacy of RAS blocker, particularly in patients with urinary protein level of 1.0 g/day or higher [8–10]. Conversely, patients with urinary protein level < 0.5 g/day have not been demonstrated adequately [11]. Our study revealed that the prescription rate of RAS blocker differed according to the amount of proteinuria (Fig. 3). These results indicated that the Japanese nephrologists generally follow the guidelines.

The use of corticosteroids is also recommended as grade IB (strong recommendation, moderate-quality evidence) based on a systematic review. In the current study, 77.8% of the respondents prescribed corticosteroids for 67% or more cases of urinary protein level > 1 g/day, as suggested in the guidelines. Although the presence of hematuria was not listed as a criterion for prescribing corticosteroids in the guidelines, the respondents emphasized on hematuria when using corticosteroids (Fig. 4a, b, and Supplementary Fig. 2). Microscopic hematuria in patients with IgAN has been identified as a strong risk factor for progression to ESKD [12, 13]. Additionally, hematuria reportedly reflects disease activity in patients with IgAN based on the multi-hit theory [14, 15]. Thus, Japanese nephrologists should consider disease activity while prescribing corticosteroids.

The adverse effects of corticosteroids include infectious diseases, worsening glycemic control, osteoporosis, and gastritis. In particular, infectious diseases during steroid administration can lead to serious complications, like life-threatening conditions. In a randomized controlled clinical trial that compared supportive care alone (in particular, RAS blocker) and in combination with immunosuppressive therapy for 3 years, severe infection was observed in the supportive care plus immunosuppressive therapy group compared with the supportive care alone group [16]. Another randomized controlled study [17] was terminated because of serious side effects, mostly infectious diseases. However, the dose of corticosteroids in these clinical trials was higher than the dose commonly used in Japan. In 2022, LV et al*.* reported the results of a clinical trial that included a group receiving a reduced corticosteroid dose [18]. Severe infection requiring hospitalization occurred in five patients in the reduced-dose group (placebo 2) compared with 12 patients in the full-dose group (placebo 1). Our results showed that there were only few cases of severe infectious diseases or serious deterioration in glycemic control. These results indicated that the Japanese nephrologists carefully checked for side effects and used the appropriate doses of corticosteroids.

Hotta et al. first reported TSP in 2001 [19]. Thereafter, several clinical studies, including randomized controlled trials, have been conducted in relation to it [20–23]. Two nationwide questionnaires, conducted in 2006 [24] and 2008 [25], indicated that TSP was the standard treatment for adult patients with IgAN in Japan. Then KDIGO guidelines described tonsillectomy would be applied only for Japanese patient with IgAN [26]. In this study, in patients with newly diagnosed IgAN, 35.8% of the respondents would perform TSP in more than 71% of patients. However, treatment protocol, especially the number of steroid pulses therapy combined with tonsillectomy, is highly variable in each institution [27, 28], and indication of TSP for elderly or child patients with IgA nephropathy are under discussion [29, 30].

The degrees of proteinuria and hematuria are commonly used as prognostic indicators during the course of treatment [13, 31–33]. In 2013, the Special IgA Nephropathy Study Group in Progressive Renal Diseases Research, affiliated with the Research on Intractable Diseases by the Ministry of Health, Labor, and Welfare in Japan, proposed new remission criteria for IgAN based on a nationwide awareness survey. Subsequently, Matsuzaki et al. reported the utility of the criteria in a single-center longitudinal cohort study [34]. In the current study, 79.5% of the respondents used the criteria to determine remission of IgAN. Since IgAN has a long-term clinical course, the concept of remission may become more important in the future.

This study had several limitations. First, it was based on a questionnaire administered to the members of the Japanese Society of Nephrology rather than a collection of actual patient data. Thus, we could not assess the clinical indicators including candidate molecules of pathogenesis of IgAN. Second, there was social-desirability bias because this questionnaire was distributed via subscribed mailing list in Japanese society of nephrology. Third, this questionnaire was intended for nephrologists resulted in possible underestimation of evidence-practice gap. Fourth, over 90% of the respondents were physicians, with only a few pediatricians. Therefore, the applicability of our results is likely limited to adult patients. To overcome these limitations, further investigation based on the nationwide-multicenter kidney disease registry included regional information, scale of facilities, and candidate molecules was needed.

## Conclusion

This questionnaire survey elucidated real-world aspects regarding the treatment for IgAN in Japan. Our findings may aid further research to determine standard treatments.

### Supplementary Information

Below is the link to the electronic supplementary material.

Supplementary file1 Supplementary Fig. 1: Important factors for use of RAS blocker. Supplementary Fig. 2: Important factors for use of corticosteroids. Supplementary Fig. 3: Important factors to determine indication of tonsillectomy with steroid pulse therapy (PPTX 58 KB)
