# Supplementary material for: Current treatment status of IgA nephropathy in Japan: a questionnaire survey
Source: Clin Exp Nephrol. 2023 Aug 30;27(12):1032–41. doi: 10.1007/s10157-023-02396-0 (PMC10654181; doi:10.1007/s10157-023-02396-0)
Supplement: Supplementary file 1 — Supplementary file1 Supplementary Fig. 1: Important factors for use of RAS blocker. Supplementary Fig. 2: Important factors for use of corticosteroids. Supplementary Fig. 3: Important factors to determine indication of tonsillectomy with steroid pulse therapy (PPTX 58 KB) [file 10157_2023_2396_MOESM1_ESM.pptx]

## Slide 1
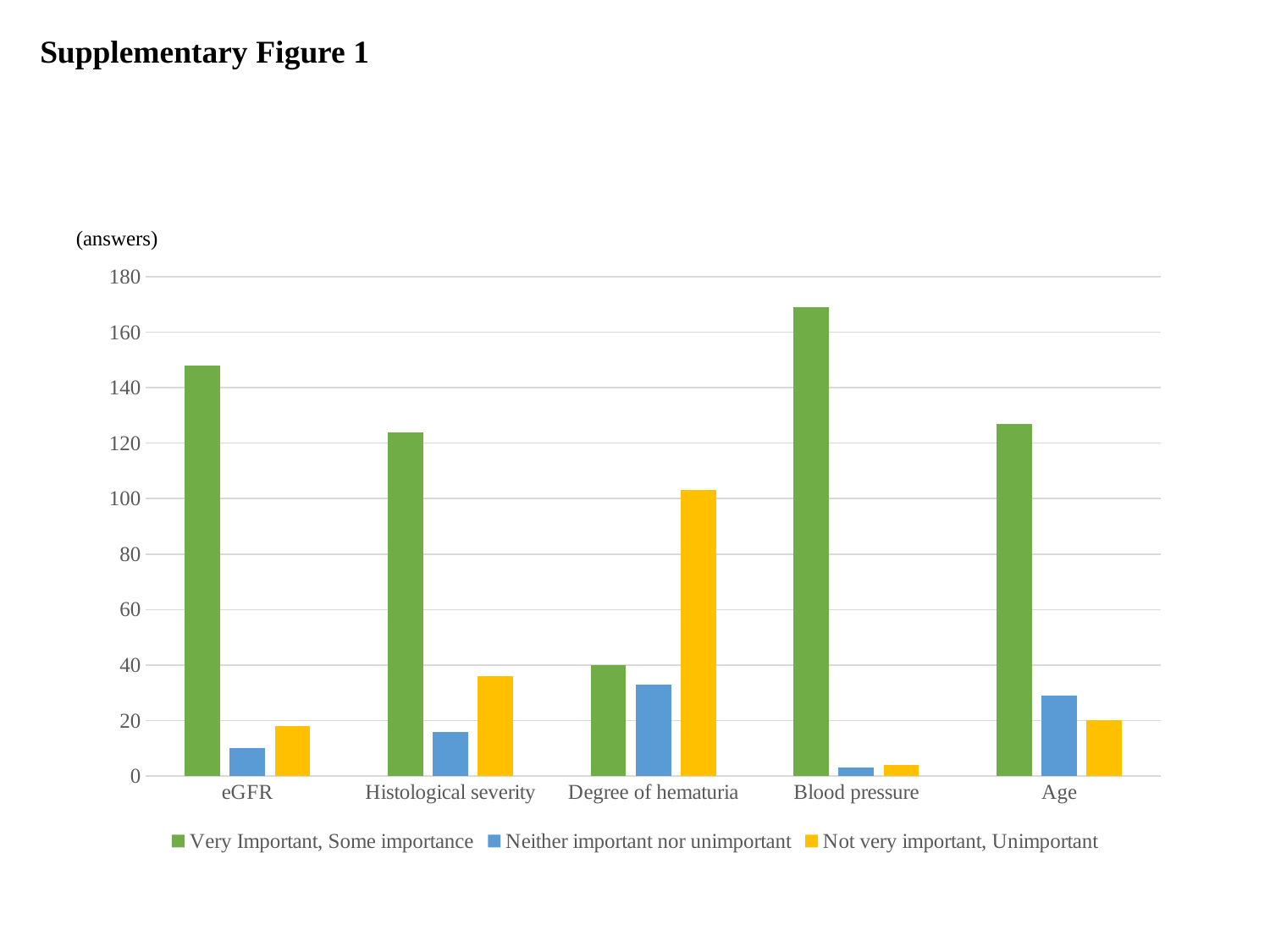

Supplementary Figure 1
(answers)
### Chart
| Category | Very Important, Some importance | Neither important nor unimportant | Not very important, Unimportant |
|---|---|---|---|
| eGFR | 148.0 | 10.0 | 18.0 |
| Histological severity | 124.0 | 16.0 | 36.0 |
| Degree of hematuria | 40.0 | 33.0 | 103.0 |
| Blood pressure | 169.0 | 3.0 | 4.0 |
| Age | 127.0 | 29.0 | 20.0 |

## Slide 2
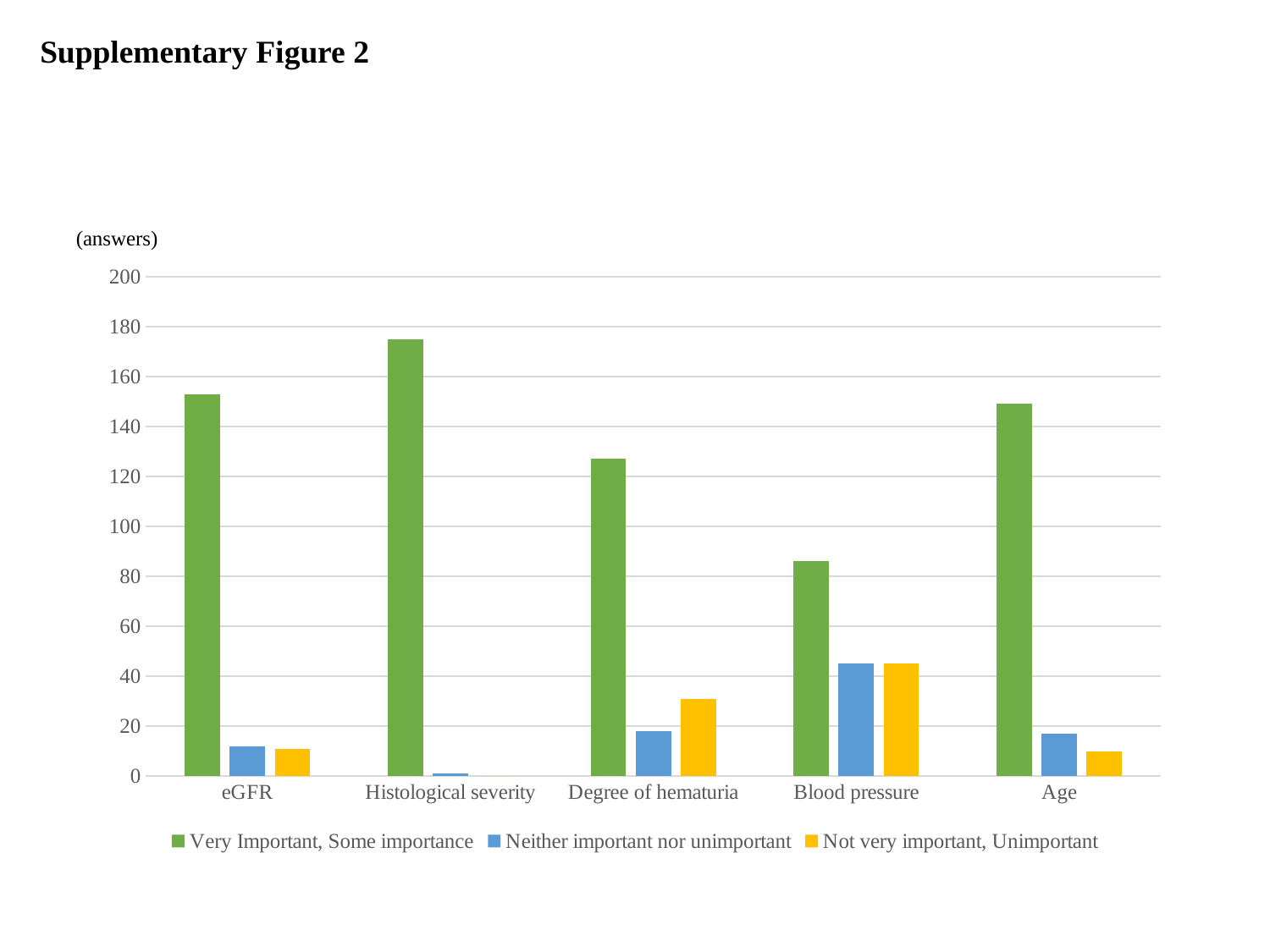

Supplementary Figure 2
(answers)
### Chart
| Category | Very Important, Some importance | Neither important nor unimportant | Not very important, Unimportant |
|---|---|---|---|
| eGFR | 153.0 | 12.0 | 11.0 |
| Histological severity | 175.0 | 1.0 | 0.0 |
| Degree of hematuria | 127.0 | 18.0 | 31.0 |
| Blood pressure | 86.0 | 45.0 | 45.0 |
| Age | 149.0 | 17.0 | 10.0 |

## Slide 3
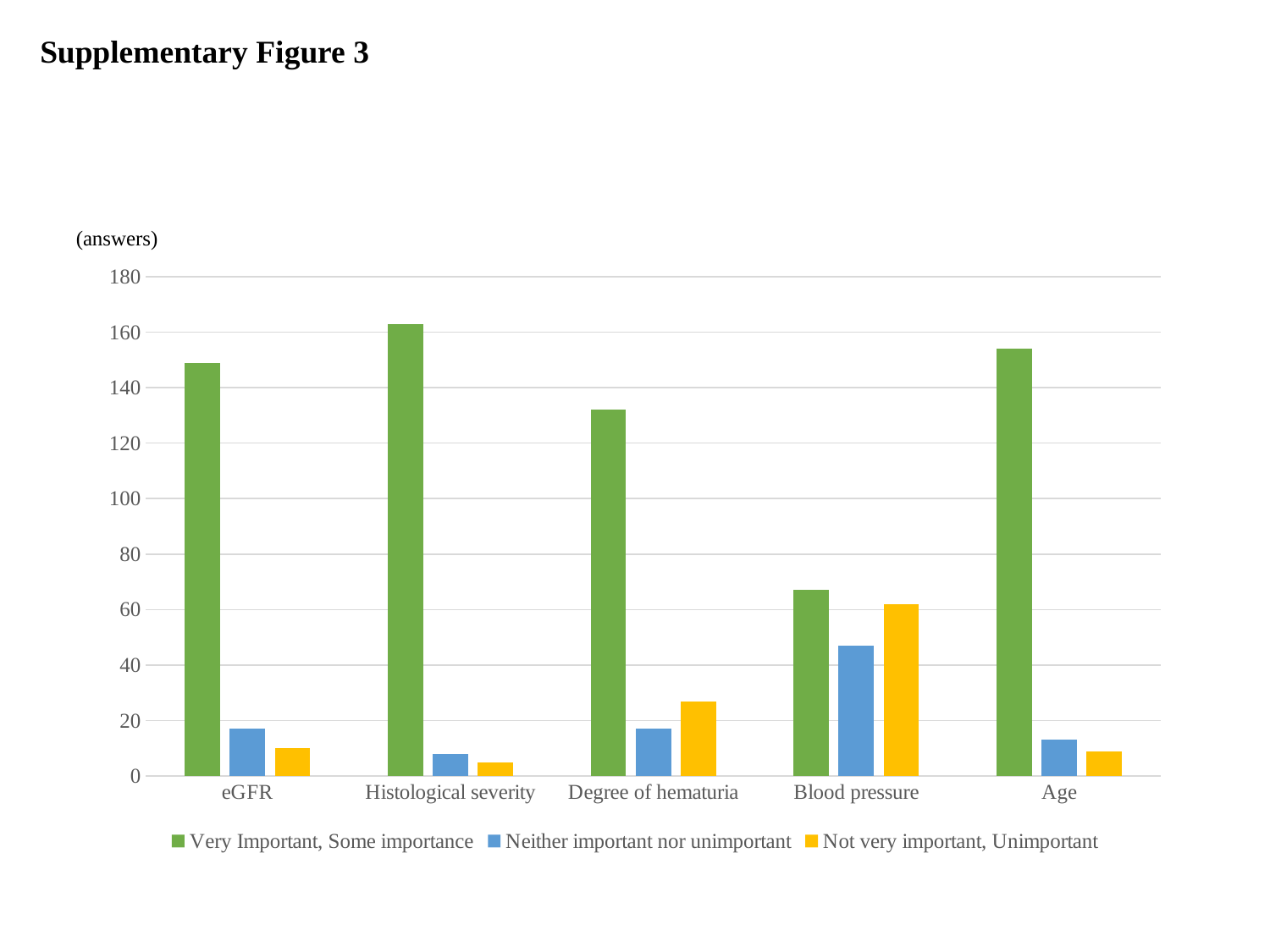

Supplementary Figure 3
(answers)
### Chart
| Category | Very Important, Some importance | Neither important nor unimportant | Not very important, Unimportant |
|---|---|---|---|
| eGFR | 149.0 | 17.0 | 10.0 |
| Histological severity | 163.0 | 8.0 | 5.0 |
| Degree of hematuria | 132.0 | 17.0 | 27.0 |
| Blood pressure | 67.0 | 47.0 | 62.0 |
| Age | 154.0 | 13.0 | 9.0 |
